# Infection of *Arabidopsis* by cucumber mosaic virus triggers jasmonate‐dependent resistance to aphids that relies partly on the pattern‐triggered immunity factor BAK1

**DOI:** 10.1111/mpp.13098

**Published:** 2021-06-22

**Authors:** Trisna Tungadi, Lewis G. Watt, Simon C. Groen, Alex M. Murphy, Zhiyou Du, Adrienne E. Pate, Jack H. Westwood, Thea G. Fennell, Glen Powell, John P. Carr

**Affiliations:** ^1^ Department of Plant Sciences University of Cambridge Cambridge UK; ^2^ NIAB EMR East Malling UK; ^3^ Institute of Bioengineering Zhejiang Sci‐Tech University Hangzhou China; ^4^ Present address: Department of Biology New York University New York New York USA; ^5^ Present address: Walder Foundation Skokie Illinois USA

**Keywords:** CMV 2b protein, epidemiology, ethylene, jasmonate, PAMP‐triggered immunity, salicylate

## Abstract

Many aphid‐vectored viruses are transmitted nonpersistently via transient attachment of virus particles to aphid mouthparts and are most effectively acquired or transmitted during brief stylet punctures of epidermal cells. In *Arabidopsis thaliana*, the aphid‐transmitted virus cucumber mosaic virus (CMV) induces feeding deterrence against the polyphagous aphid *Myzus persicae*. This form of resistance inhibits prolonged phloem feeding but promotes virus acquisition by aphids because it encourages probing of plant epidermal cells. When aphids are confined on CMV‐infected plants, feeding deterrence reduces their growth and reproduction. We found that CMV‐induced inhibition of growth as well as CMV‐induced inhibition of reproduction of *M*. *persicae* are dependent upon jasmonate‐mediated signalling. BRASSINOSTEROID INSENSITIVE1‐ASSOCIATED KINASE1 (BAK1) is a co‐receptor enabling detection of microbe‐associated molecular patterns and induction of pattern‐triggered immunity (PTI). In plants carrying the mutant *bak1‐5* allele, CMV induced inhibition of *M*. *persicae* reproduction but not inhibition of aphid growth. We conclude that in wildtype plants CMV induces two mechanisms that diminish performance of *M*. *persicae*: a jasmonate‐dependent and PTI‐dependent mechanism that inhibits aphid growth, and a jasmonate‐dependent, PTI‐independent mechanism that inhibits reproduction. The growth of two crucifer specialist aphids, *Lipaphis erysimi* and *Brevicoryne brassicae*, was not affected when confined on CMV‐infected *A. thaliana*. However, *B*. *brassicae* reproduction was inhibited on CMV‐infected plants. This suggests that in *A. thaliana* CMV‐induced resistance to aphids, which is thought to incentivize virus vectoring, has greater effects on polyphagous than on crucifer specialist aphids.

## Graphical abstract

Cucumber mosaic virus (CMV)‐induced BRASSINOSTEROID INSENSITIVE1‐ASSOCIATED KINASE1‐ and jasmonic acid (JA)‐dependent resistance repels the generalist *Myzus* *persicae*, enhancing CMV transmission; only JA‐dependent resistance affects the specialist *Brevicoryne* *brassicae*; *Lipaphis* *erysimi* is unaffected by either mechanism.

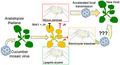

## 1 INTRODUCTION

Aphids, whiteflies, or other phloem‐feeding insects vector most plant viruses (Bragard et al., 2013; Canto et al., 2009; Carr et al., 2019; Jones, 2014). Cucumber mosaic virus (CMV) can infect over 1,000 plant species, and one of these is the important experimental model, *Arabidopsis thaliana* (Hily et al., 2014; Pagán et al., 2010; Yoon et al., 2019). CMV can be vectored nonpersistently by at least 60 species of aphids, including the well‐studied peach‐potato or green peach aphid (*Myzus persicae*), a polyphagous aphid that exploits a wide range of plants for nutrition (Kennedy et al., 1962; Krenz et al., 2015; Nalam et al., 2019). Virions of nonpersistently transmitted viruses bind loosely to receptors in the aphid stylet and do not circulate within the aphid body. Therefore, CMV and other nonpersistently transmitted viruses are most efficiently acquired and transmitted during brief probes of aphid stylets into plant epidermal cells (Krenz et al., 2015; Liang & Gao, 2017; Powell, 2005; Tjallingii et al., 2010; Webster et al., 2017, 2018).

Several aphid‐transmitted viruses have been shown to modify the metabolism or defence status of their plant hosts in ways that affect aphid visitation and feeding (Carmo‐Sousa et al., 2014; Casteel et al., 2014, 2015; Chesnais et al., 2019; Hodge & Powell, 2008, 2010; Mauck, 2016; Mauck et al., 2010, 2019; Nalam et al., 2019; Tungadi et al., 2020; Wamonje et al., 2020a, 2020b; Westwood et al., 2013; Ziebell et al., 2011). It has been suggested that these virus‐induced changes in aphid–plant interactions may promote virus acquisition and transmission (Carr et al., 2019; Groen et al., 2017; Mauck, 2016; Mauck et al., 2010, 2019). Epidemiological modelling indicates that if viruses induce emission of attractive volatile organic compounds from the host plant accompanied by factors that deter aphids from prolonged phloem feeding and plant colonization, this will encourage virus acquisition and accelerate dispersal of inoculum to plants in the immediate vicinity of the infected host (Donnelly et al., 2019).

CMV infection induces feeding deterrence against *M*. *persicae* in plants of the Col‐0 accession of *A. thaliana*, as well as in cucurbits and common bean (*Phaseolus vulgaris*) (Mauck et al., 2010; Wamonje et al., 2020a; Westwood et al., 2013). Electronic monitoring of aphid feeding behaviour showed that aphids placed on CMV‐infected plants were deterred from feeding on phloem tissue but not from probing epidermal cells (Wamonje et al., 2020a; Westwood et al., 2013). Additionally, aphids confined on CMV‐infected plants of *A. thaliana* grow poorly (Westwood et al., 2013). It was found by Westwood et al. (2013) that CMV‐induced feeding deterrence in *A. thaliana* was mediated by increased biosynthesis and accumulation of 4‐methoxy‐indol‐3‐yl‐methylglucosinolate (4MI3M), especially around the phloem tissue. The glucosinolate 4MI3M is a metabolite that aphids find distasteful (Kim et al., 2008). The 2a protein, one of five proteins encoded by CMV, is responsible for induction of feeding deterrence and concomitant growth inhibition, and this mechanism predominates during infection over a stronger anti‐aphid resistance mechanism due to direct and indirect interactions between two other viral proteins (the 1a and 2b proteins), and the plant Argonaute 1 protein (Rhee et al., 2020; Watt et al., 2020; Westwood et al., 2013). CMV infection induces expression of several genes known to be responsive to microbe‐associated molecular pattern (MAMP) molecules (Westwood et al., 2013). Furthermore, 4MI3M accumulation is increased in plants exhibiting pattern‐triggered immunity (PTI) (Clay et al., 2009). The primary function of the CMV 2a protein is to act as the viral RNA‐dependent RNA polymerase, but one of its additional activities appears to be to stimulate feeding deterrence via PTI activation and thereby accelerate aphid‐mediated virus transmission (Westwood et al., 2013). However, not all aphids are as repelled as *M*. *persicae*, a nonspecialist polyphagous aphid, by 4MI3M or other glucosinolates. For example, *Lipaphis erysimi* (mustard aphid) and *Brevicoryne brassicae* (cabbage aphid) are crucifer specialists that accumulate glucosinolates in their bodies to act as defences or deterrents against their natural enemies (Blackman & Eastop, 2000; Blande et al., 2007; Kazana et al., 2007). At the start of this study, it was not known how crucifer specialist aphids would respond to CMV‐induced changes in the metabolism or defence status of *A. thaliana*.

PTI is an important line of defence against bacterial, fungal, and oomycete pathogens (Chinchilla et al., 2007, 2009) and functions in protection against aphids and nematodes (Prince et al., 2014; Teixeira et al., 2016). BRASSINOSTEROID INSENSITIVE1‐ASSOCIATED KINASE1 (BAK1) is a plant co‐receptor molecule that enables detection of several MAMPs by pattern‐recognition receptors and activation of PTI (Chinchilla et al., 2007, 2009). Interestingly, BAK1, BAK1‐LIKE (BKK1), and other signalling components of PTI in *A. thaliana* and tomato play roles in limiting the accumulation of several viruses, and certain viral proteins have been shown to inhibit PTI, suggesting that PTI is also involved in antiviral defence (Kørner et al., 2013; Nicaise & Candresse, 2017; Niehl et al., 2016; Yang et al., 2010; Zorzatto et al., 2015). In this work we explored the extent to which induction by CMV of feeding deterrence and concomitant growth inhibition is dependent on the activity of BAK1 and the operation of key defensive signal transduction pathways. We also investigated how specialist aphids respond to CMV‐infected *A. thaliana*.

## 2 RESULTS

### 2.1 CMV infection induces at least two mechanisms that constrain *M*. *persicae* performance on *A. thaliana*

**FIGURE 1:**
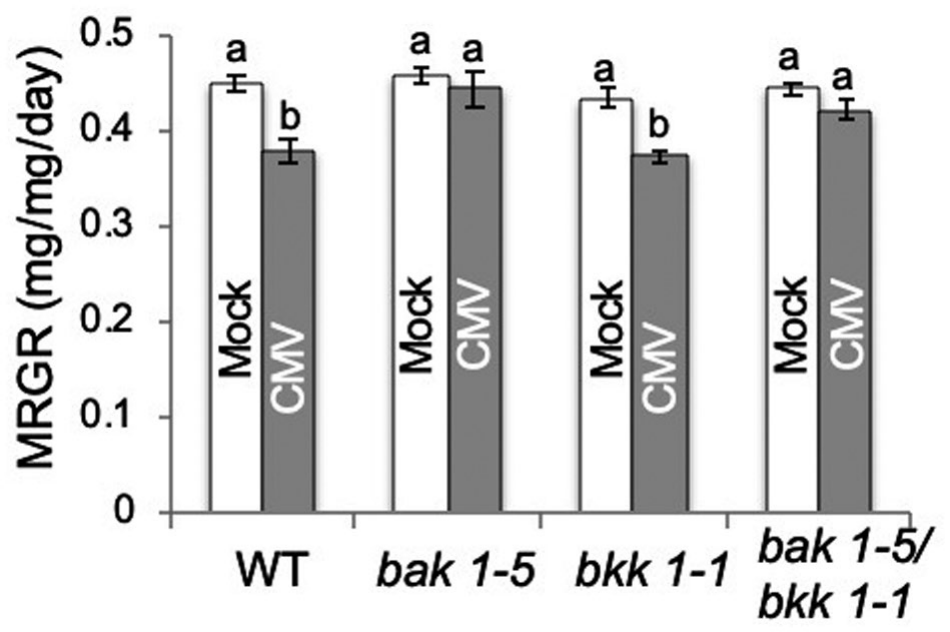
Cucumber mosaic virus (CMV)‐induced resistance to aphid growth on *Arabidopsis thaliana* plants requires *BAK1* but not *BKK1*. The mean relative growth rate (MRGR) of aphids (*Myzus persicae*) placed on wildtype Col‐0 (WT), *bak1‐5*, *bkk1‐1*, or *bak1‐5/bkk1‐1* mutant plants that had been inoculated with CMV or mock‐inoculated with sterile water. One‐day‐old nymphs (*n* = 7–20 per treatment) were weighed, confined on plants for 5 days, and reweighed. Statistically significantly differences between MRGR values are indicated by different letters (a, b: analysis of variance with post hoc Tukey's HSD test, *p* < .05). Error bars indicate standard error around the mean

Previous work suggested a role for PTI in CMV‐induced aphid feeding deterrence, and the concomitant decrease in mean relative growth rate (MRGR) observed for aphids (*M*. *persicae*) confined on infected *A. thaliana* plants (Westwood et al., 2013). BAK1 and BKK1 are co‐receptors involved in responses that follow perception of several MAMPs (Chinchilla et al., 2007; Heese et al., 2007). Therefore, we investigated whether BAK1 and/or BKK1 are required for CMV‐induced feeding deterrence using plants carrying the mutant alleles *bak1‐5* and *bkk1‐1*. The *bak1‐5* allele carries a point mutation at a single amino acid residue that impairs the ability of plants to perceive MAMPs (e.g., flg22) but does not affect BAK1’s roles in cell death regulation or brassinosteroid signalling (Roux et al., 2011; Schwessinger et al., 2011). *M*. *persicae* growth rates were not decreased on CMV‐infected *bak1‐5* or *bak1‐5/bkk1‐1* double‐mutant plants (Figure 1 and Spreadsheet S1), showing that BAK1 contributes to induction of aphid resistance in *A. thaliana*. However, the CMV‐induced inhibition of aphid growth was observed for *M*. *persicae* confined on *bkk1‐1* mutant plants (Figure 1). Thus, it appears that BAK1, but not BKK1, plays a role in facilitating CMV‐induced inhibition of aphid growth on *A. thaliana*.

**FIGURE 2:**
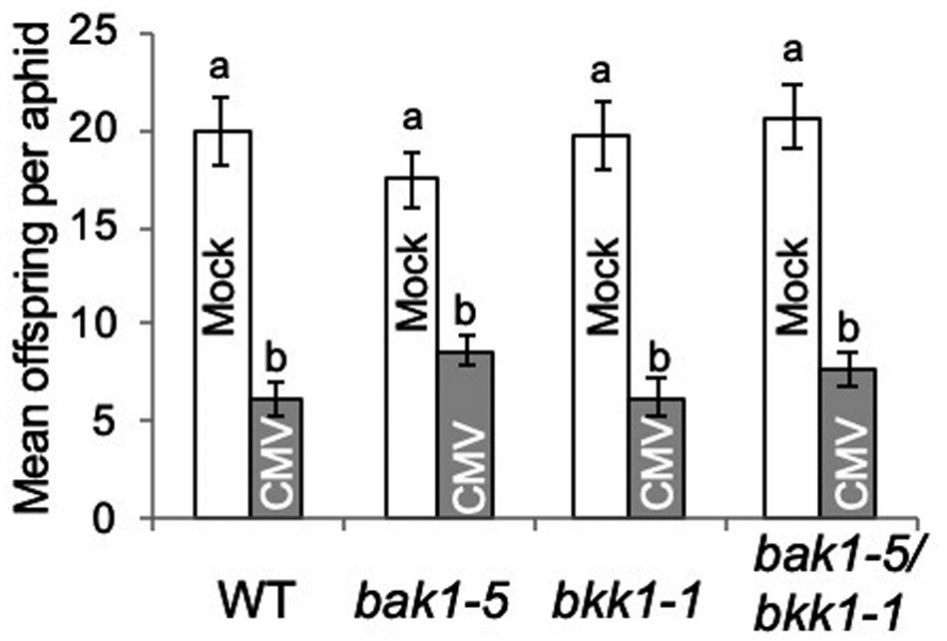
Cucumber mosaic virus (CMV)‐induced resistance to aphid reproduction on *Arabidopsis thaliana* is not dependent upon the *BAK1* or *BKK1* genes. One‐day‐old aphid (*Myzus persicae*) nymphs (*n* = 10 per treatment) were confined for 14 days on *Arabidopsis thaliana* Col‐0 wildtype (WT), or *bak1‐5*, *bkk1‐1*, or *bak1‐5/bkk1‐1* mutant plants, which had been inoculated with CMV or mock‐inoculated with sterile water, and offspring counted. Mean offspring produced per aphid was calculated for each treatment. Error bars indicate standard error around the mean, and bars with different letters indicate statistically significant differences in offspring production (analysis of variance with post hoc Tukey's HSD test, *p* < .05)

We recently showed that in *A. thaliana* the CMV 2a protein induces resistance not only to aphid growth but also to aphid reproduction (Rhee et al., 2020). Here, we confirmed that the fecundity of aphids placed on CMV‐infected plants was diminished compared to aphids placed on mock‐inoculated plants (Figure 2 and Spreadsheet S1). Interestingly, and in contrast to our results for CMV‐induced inhibition of aphid growth (Figure 1), the CMV‐induced decrease in aphid fecundity was not abolished on *bak1‐5* or *bak1‐5/bkk1‐1* double‐mutant plants (Figure 2). Thus, CMV infection induces at least two aphid resistance mechanisms in *A. thaliana*. One inhibits growth of individual aphids and is BAK1‐dependent (Figure 1). The other mechanism decreases the ability of aphids to reproduce but its induction by CMV does not require BAK1 (Figure 2). CMV accumulated to similar levels in the wildtype and *bak1‐5* and *bkk1‐1* mutant plants (Figure S1). Thus, although CMV infection can trigger a BAK1‐dependent response (induction of feeding deterrence against aphids), neither BAK1 nor BKK1 appear to condition basal resistance against CMV, which contrasts with the roles of these co‐receptors in maintaining basal resistance against several other viruses (Kørner et al., 2013; Nicaise & Candresse, 2017; Niehl et al., 2016; Yang et al., 2010; Zorzatto et al., 2015).

### 2.2 Jasmonic acid is required for CMV‐induced inhibition of *M. persicae* growth and reproduction

**FIGURE 3:**
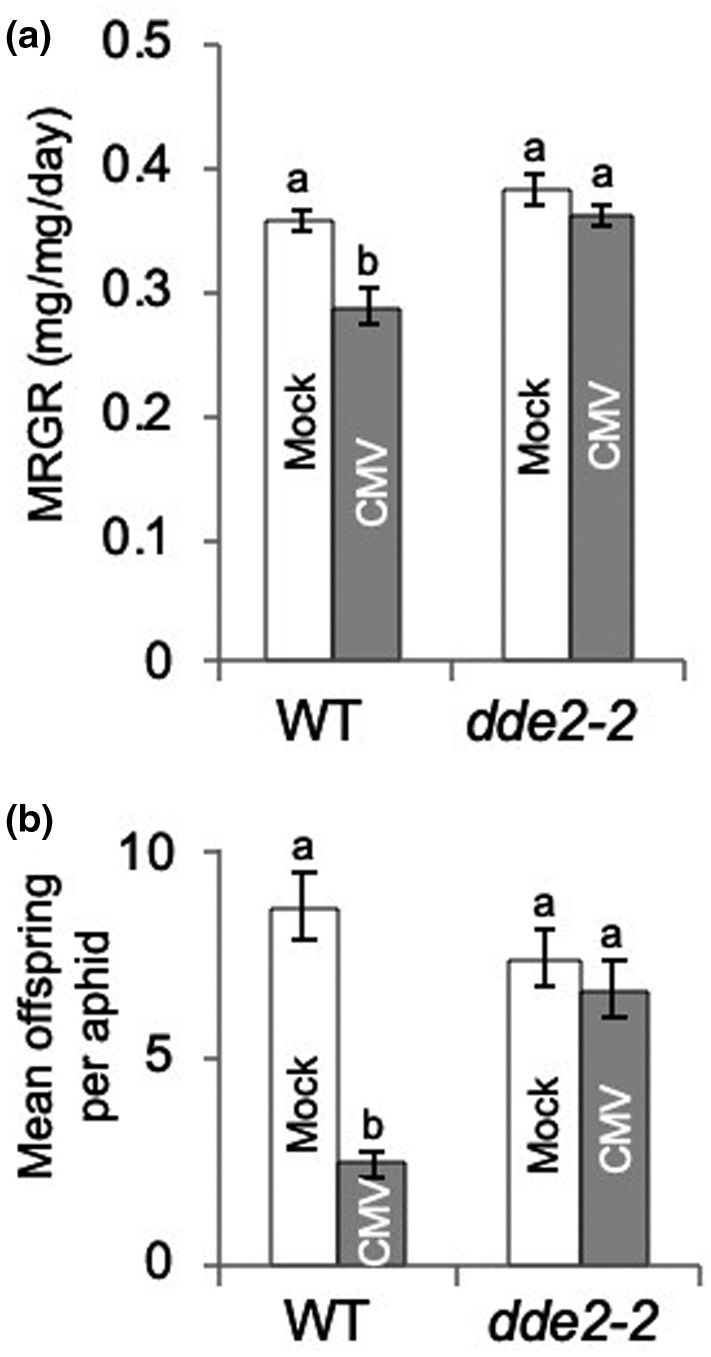
Cucumber mosaic virus (CMV)‐induced resistance to aphids is abolished on plants of the *dde2‐2* mutant line. (a) Mean relative growth rate (MRGR) of aphid (*Myzus persicae*) nymphs placed on *Arabidopsis thaliana* (Col‐0) wildtype (WT) plants and *dde2‐2* (mock‐inoculated or inoculated with CMV) was determined by weighing 1‐day‐old aphids at the time of placement and 6 days later (*n* = 16–20 aphids per treatment) before replacing on the plants. (b) At 14 days after placement the numbers of offspring produced by each aphid were counted and mean offspring per aphid calculated. Error bars indicate standard error around the mean, and bars with different letters indicate statistically significant differences in MRGR (a) or reproduction (b) (analysis of variance with post hoc Tukey's HSD test, *p* < .05)

**FIGURE 4:**
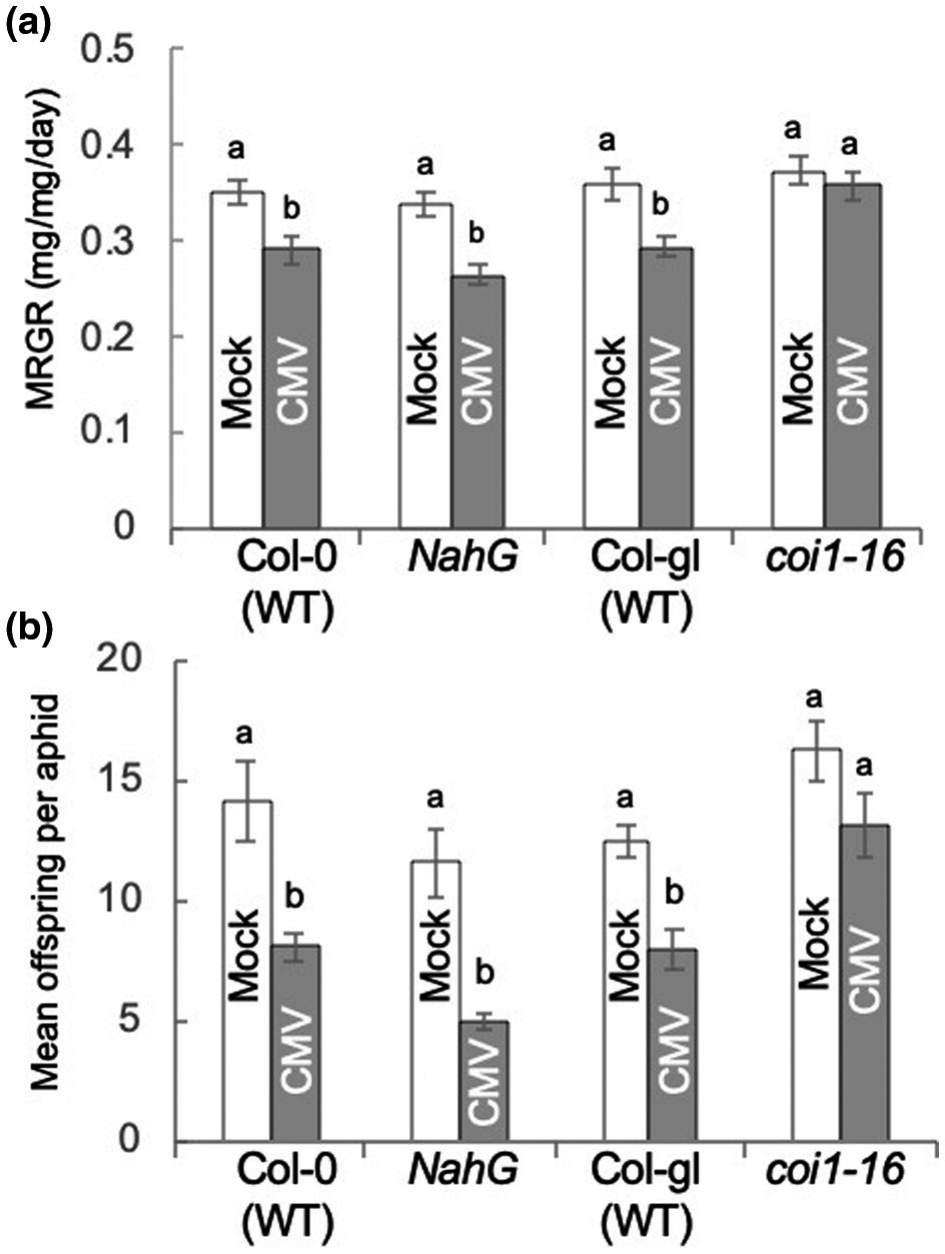
Aphid performance on cucumber mosaic virus (CMV)‐infected *NahG*‐transgenic and *coi1‐16* mutant *Arabidopsis thaliana* plants. (a) Mean relative growth rates (MRGRs) of aphids (*Myzus persicae*) confined on *NahG*‐transgenic and *coi1‐16* mutant plants were compared with those for aphids on the respective untransformed, wildtype (WT) accessions of *Arabidopsis thaliana*, Col‐0 and Col‐gl (*n* = 13–20 aphids per treatment). Plants had been previously mock‐inoculated or infected with CMV. One‐day‐old nymphs were weighed before placement on plants, reweighed 6 days later, and replaced on plants. (b) At 14 days after placement offspring were counted and the mean number of offspring produced per aphid calculated. Error bars indicate standard error around the mean, and bars with different letters indicate statistically significant differences in MRGR (a) or reproduction (b) (analysis of variance with post hoc Tukey's HSD test, *p* < .05)

Jasmonic acid (JA) is a phytohormone that has roles in insect resistance (Meldau et al., 2011; Vos et al., 2013). Using mutant lines compromised in JA biosynthesis (*delayed dehiscence 2‐2*: *dde2‐2*) or JA perception (*coronatine insensitive 1‐16*: *coi1‐16*), we found that both CMV‐induced aphid resistance mechanisms in *A. thaliana* are dependent on this phytohormone. In contrast to the effect of CMV in wildtype plants, there was no reduction in either growth rate or fecundity for aphids confined on CMV‐infected *dde2‐2* (Figure 3 and Spreadsheet S2) or *coi1‐16* mutant plants (Figure 4 and Spreadsheet S3). Hence, JA‐dependent signalling is required not only for BAK1‐dependent induction of resistance to aphid growth but also for the induction of resistance to aphid colony growth.

**FIGURE 5:**
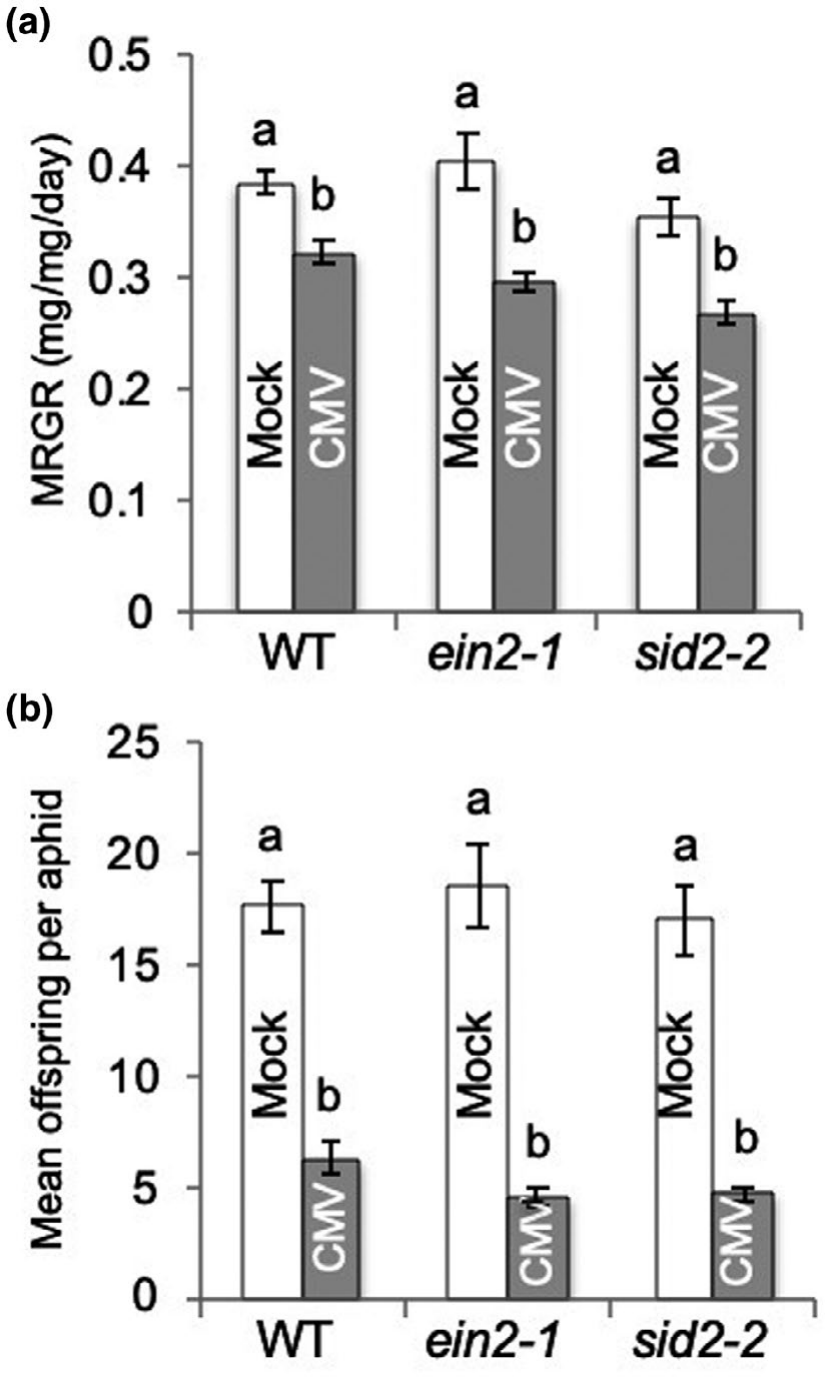
Aphid performance on cucumber mosaic virus (CMV)‐infected *ein2* and *sid2* mutant *Arabidopsis thaliana* plants. (a) The mean relative growth rate (MRGR) of aphids (*Myzus persicae*) was compared on wild‐type (WT) *Arabidopsis thaliana* Col‐0 plants versus plants of the mutant lines *sid2‐2* and *ein2‐1* (*n* = 18–24 aphids per treatment). Plants had been previously either mock‐inoculated or infected with CMV. One‐day‐old nymphs were weighed before placement on plants, reweighed 6 days later, and replaced on plants. (b) At 14 days after placement offspring were counted and mean number of offspring produced per aphid calculated. Error bars indicate standard error around the mean, and bars with different letters indicate statistically significant differences in MRGR (a) or reproduction (b) (analysis of variance with post hoc Tukey's HSD test, *p* < .05)

Aphids were confined on transgenic plants unable to accumulate salicylic acid (SA) (*NahG*‐transgenic) (Figure 4) or on mutant plants compromised in SA biosynthesis (*salicylic acid induction deficient 2‐2: sid2‐2*) (Figure 5 and Spreadsheet S4). For both SA‐depleted lines, *M*. *persicae* MRGR and colony growth were decreased on CMV‐infected plants, as they were on CMV‐infected nontransgenic and wildtype plants (Figures 4 and 5). ETHYLENE INSENSITIVE2 (EIN2) is a membrane protein required for ethylene signalling (Alonso et al., 1999). Loss of EIN2 function is known to abolish the virus‐induced susceptibility to *M*. *persicae* observed in *A. thaliana* infected with turnip mosaic virus (Casteel et al., 2015). However, in plants carrying the *ein2‐1* mutant allele, CMV infection engendered decreases in aphid MRGR and aphid fecundity (Figure 5). Therefore, neither of the defensive phytohormones SA or ethylene is required for CMV‐induced resistance to aphid growth or reproduction in *A. thaliana*.

### 2.3 CMV‐induced effects on the performance of crucifer‐specialist aphids

**FIGURE 6:**
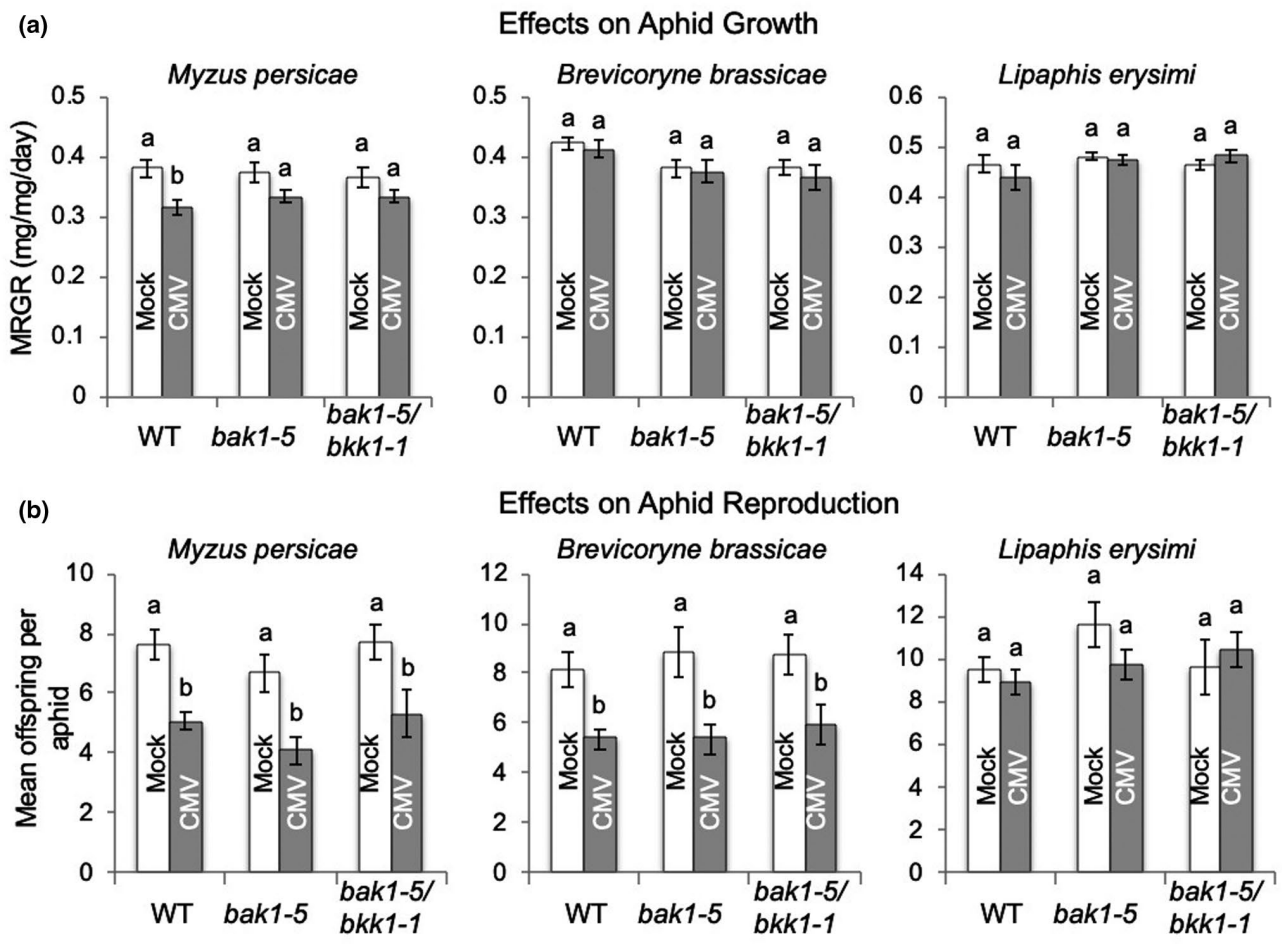
Performance of generalist and crucifer‐specializing aphids on cucumber mosaic virus (CMV)‐infected *Arabidopsis thaliana* plants carrying defective copies of the *BAK1* gene. (a) The mean relative growth rates (MRGR) of aphids of a generalist (*Myzus persicae*) and two crucifer specialist species (*Brevicoryne brassicae* and *Lipaphis erysimi*) were measured on wildtype (WT) *Arabidopsis thaliana* Col‐0 plants and plants of the mutant line *bak1‐5* and the double‐mutant *bak1‐5/bkk1‐1* (*n* = 10–23 aphids per treatment). Plants had been previously either mock‐inoculated or infected with CMV. One‐day‐old nymphs were weighed before placement on plants, reweighed 6 days later, and replaced on plants. (b) At 14 days after placement offspring were counted and the mean number of offspring produced per aphid calculated. Error bars indicate standard error around the mean, and bars with different letters indicate statistically significant differences in MRGR (a) or reproduction (b) (analysis of variance with post hoc Tukey's HSD test, *p* < .05)

The oligophagous aphids *L*. *erysimi* and *B*. *brassicae* specialize on crucifers and are less affected by these plants’ chemical defences than the polyphagous *M*. *persicae* (Blackman & Eastop, 2000; Blande et al., 2007; Fening et al., 2020; Kazana et al., 2007). We hypothesized that the performance of crucifer specialists might be less affected than that of the generalist aphid, *M*. *persicae*, by CMV‐induced changes in the metabolism or defence status of *A. thaliana*. Neither the growth nor the reproduction of *L*. *erysimi* was affected when aphids of this species were confined on CMV‐infected plants (Figure 6 and Spreadsheet S5). For *B*. *brassicae* confined on CMV‐infected plants, the growth of these aphids was unaffected (Figure 6a). However, their reproduction was significantly decreased (Figure 6b). CMV‐induced inhibition of *B*. *brassicae* reproduction was, as for CMV‐induced inhibition of *M*. *persicae* reproduction, not dependent upon BAK1 (Figures 2 and 6). The results confirm that in *A. thaliana* CMV induces two distinct mechanisms that diminish aphid performance and show that whereas *M*. *persicae* is affected by both mechanisms, *B*. *brassicae* is affected by only one (inhibition of reproduction) and *L*. *erysimi* is unaffected by either mechanism.

## 3 DISCUSSION

During CMV infection of *A. thaliana* the CMV 2a protein stimulates at least two forms of resistance to aphids: inhibition of aphid growth and inhibition of aphid reproduction (Rhee et al., 2020; Westwood et al., 2013). We have shown that the inhibition of growth is dependent on BAK1, consistent with our previously suggested role for PTI, whilst the inhibition of aphid reproduction is BAK1‐independent (Figure 2). Nevertheless, both mechanisms require JA‐dependent defensive signalling to function but neither depend on SA‐ or ethylene‐mediated defensive signalling (Figures 3, 4, 5). Both of these CMV‐stimulated anti‐aphid resistance mechanisms affect the polyphagous aphid *M*. *persicae*. Of the two crucifer‐adapted aphids we examined, *L*. *erysimi* is affected by neither of the CMV‐induced anti‐aphid resistance mechanisms, while *B*. *brassicae* is unaffected by CMV‐induced inhibition of aphid growth but is inhibited in its ability to reproduce on CMV‐infected plants (Figure 6). *B*. *brassicae* and *L*. *erysimi* are tolerant of glucosinolates and can store them, in contrast to *M*. *persicae*, which is less tolerant of these crucifer‐specific compounds (Blande et al., 2007). The results are consistent with those of Westwood et al. (2013), who showed that accumulation of the glucosinolate 4MI3M in vascular tissue inhibits phloem feeding by *M*. *persicae*, which leads to decreased growth of these aphids when confined on CMV‐infected *A. thaliana* plants. However, they also show that an additional CMV‐induced mechanism (or mechanisms) must be at play that causes decreased fecundity of *M*. *persicae* and *B*. *brassicae*, but to which *L*. *erysimi* is immune.

Under natural conditions, when aphids are not confined on CMV‐infected plants, induction of feeding deterrence or of other forms of resistance will inhibit settling and encourage dispersal of virus‐bearing *M*. *persicae* away from CMV‐infected *A. thaliana* plants. This is likely to promote transmission of CMV to immediately neighbouring uninfected plants (Donnelly et al., 2019). Our results would suggest that the tendency of *L*. *erysimi* aphids to settle on or disperse from *A. thaliana* would not be affected by this host's CMV infection status. The probable effect on *B*. *brassicae* is less clear. If *B*. *brassicae* aphids are not deterred from settling on CMV‐infected plants, this will decrease their reproductive fitness. Understanding definitively how these differing effects of CMV‐induced aphid resistance on aphid dispersal and CMV transmission by the nonspecialist *M*. *persicae* compares with the two specialists will require additional studies, including free‐choice assays in which aphids are free to move between plants and transmit virus.

In the meantime, however, we must consider if the effects of host infection status on crucifer‐specialist aphids are likely to have epidemiological relevance. In nature, *L*. *erysimi* is an effective vector for CMV, although not as efficient as *M*. *persicae* (Berlandier et al., 1997; Tian et al., 2012). However, while *B*. *brassicae* is associated with transmission of crucifer‐specialist viruses such as cauliflower mosaic virus and turnip yellows virus (Chesnais et al., 2019; Moreno et al., 2005), it has been reported to be a nonvector for CMV (Kennedy et al., 1962). For the present, we conclude that it may be more beneficial for CMV transmission that the performance of polyphagous aphids is diminished on CMV‐infected *A. thaliana* plants because it incentivizes them to transmit the virus to neighbouring host plants. An advantage of this for CMV is that because it has a very wide host range, a polyphagous vector is more likely to deliver it to a suitable host than a specialist aphid. However, it is not a general rule that only nonspecialist aphids can be encouraged to enhance virus transmission through virus‐induced modification of host–aphid interactions. Recently, we showed that CMV, as well as the potyviruses bean common mosaic virus and bean common mosaic necrosis virus, induce feeding deterrence in common bean against the bean specialist *Aphis* *fabae*, as well as against the generalist *M*. *persicae*, and that consequent changes in the feeding behaviour of both aphids were likely to enhance onward transmission of all three viruses by both vectors (Wamonje et al., 2020a).

Biosynthesis of the aphid feeding deterrent 4MI3M by *A. thaliana* is stimulated by the PTI signalling network (Clay et al., 2009; Mewis et al., 2005, 2006). Previous work showed that CMV‐induced activation of PTI‐related signalling and increased 4MI3M biosynthesis explained to a large extent the decrease in growth rates of *M*. *persicae* placed on infected plants (Westwood et al., 2013). However, aphid growth and fecundity assays with *bak* *1* mutant plants, and contrasts between the responses of different aphid species, indicate that the inhibition of *M*. *persicae* reproduction on CMV‐infected plants is not induced in the same way as growth inhibition, and may not involve 4MI3M. Instead, inhibition of reproduction may require increased synthesis of one or more other anti‐insect plant metabolites to which *M*. *persicae* and *B*. *brassicae* are sensitive, but *L*. *erysimi* is not. Previous work on interactions between *A. thaliana* and *B*. *brassicae* showed that this aphid is sensitive to camalexin (Kuśnierczyk et al., 2008). However, accumulation of this compound is not significantly increased by CMV infection (Westwood et al., 2013). Currently, therefore, it is not clear what metabolite(s) might be likely to be responsible for the inhibition of reproduction by *M*. *persicae* and *B*. *brassicae* on CMV‐infected *A. thaliana* plants.

Although CMV infection stimulates PTI, no marked increases in CMV accumulation were seen in *bak1* mutant plants (Figure S1), which contrasts with work with certain other viruses, where a mutation in BAK1 will result in increased virus accumulation, showing that PTI plays a role in limiting their multiplication (Kørner et al., 2013; Nicaise & Candresse, 2017; Niehl et al., 2016; Yang et al., 2010; Zorzatto et al., 2015). Thus, CMV appears to be able to trigger a potent resistance response, which is useful for modifying the interactions of its host with its vector, without suffering any consequences for its ability to replicate or spread. It is possible to speculate that another viral protein might “protect” CMV from induction of PTI by the 2a protein. For example, the CMV 3a movement protein has been reported to have inhibitory effects on PTI (Kong et al., 2018). Interestingly, CMV has been shown in some hosts to be able to induce another resistance mechanism (the hypersensitive response) without being limited in its spread by the programmed death of the initially infected host cells (Kim & Palukaitis, 1997; Tian et al., 2020). Perhaps related to its ability to exploit one of the largest host ranges of any virus (Yoon et al., 2019), CMV has adapted to not only evade a wide range of resistance mechanisms, but also to exploit them.

We found, using *coi* *1* and *dde* *2* mutant plants, that JA‐mediated signalling is necessary for both forms of CMV‐induced aphid resistance, whereas SA‐mediated and ethylene‐mediated signalling are not required (Figures 3, 4, 5). This is consistent with findings regarding other three‐way pathogen–plant–insect interactions, where JA has proved to be a key signal. Examples include interactions of the bacterial phytopathogen *Pseudomonas syringae* and the chewing herbivores *Scaptomyza flava* and *Trichoplusia ni* with *A. thaliana* (Groen et al., 2013, 2016); the aster yellows phytoplasma and its leafhopper vector *Macrosteles quadrilineatus* in *A. thaliana* (Sugio et al., 2011), and persistently transmitted begomoviruses with their *Bemisia tabaci* vector and their plant hosts (Li et al., 2019; Sun et al., 2017; Zhang et al., 2012). The results are also consistent with previous work showing that CMV and its 2b protein can inhibit induction of JA‐mediated gene expression (Lewsey et al., 2010; Westwood et al., 2014; Wu et al., 2017). Sequences within the CMV 2a protein (Rhee et al., 2020) trigger the induction of both the mechanism that inhibits growth of *M*. *persicae* (which we have shown here to be BAK1‐dependent), as well as the mechanism that inhibits reproduction of *M*. *persicae* and *B*. *brassicae* (which we have shown here to be BAK1‐independent). Taken together, these results suggest that the 2a protein most likely interferes in some manner with JA‐mediated signalling, and that this leads to induction of at least two mechanisms that inhibit aphid performance on *A. thaliana*.

## 4 EXPERIMENTAL PROCEDURES

### 4.1 Plant and virus materials

Seeds of *A. thaliana* accessions Col‐0 and Col‐gl were obtained from the Nottingham Arabidopsis Stock Centre. Mutant alleles were in the Col‐0 background unless indicated otherwise. The *NahG* transgenic line and the *dde2‐2*, *ein2‐1* , *sid2‐2*, *coi1‐16*, *bak1‐5*, and *bkk1‐1* mutants and *bak1‐5*/*bkk1‐1* double‐mutant line have all been characterized previously (Albrecht et al., 2008; Alonso et al., 1999; Bartsch et al., 2006; Delaney et al., 1994; Ellis & Turner, 2002; Guzman & Ecker, 1990; Heese et al., 2007; Kemmerling et al., 2007; von Malek et al., 2002; Schwessinger et al., 2011; Westphal et al., 2008; Wildermuth et al., 2001; Xie et al., 1998). Seeds of Chinese cabbage (*Brassica rapa* var. *pekinensis* ‘Green Rocket’) were obtained from Tozer Seeds and plants were used to maintain aphid colonies. Tobacco (*Nicotiana tabacum* ‘Xanthi‐nc’) and *Nicotiana benthamiana* were used for virus propagation. Plant growth conditions have been described previously (Lewsey et al., 2009, 2010; Westwood et al., 2013).

### 4.2 Virus purification and inoculation

Virions of Fny‐CMV (Roossinck & Palukaitis, 1990) were purified from tobacco or *N*. *benthamiana* as described by Palukaitis (2019). Virions (100 μg/ml in sterile water) were mechanically inoculated onto carborundum‐dusted leaves of wildtype, mutant or transgenic *A. thaliana* plants at the four‐ to six‐true‐leaf stage. Mock inoculation used sterile water only. Infection was confirmed using double antibody sandwich enzyme‐linked immunosorbent assay kits (Bioreba) with absorbance at 405 nm measured using a Titertek Multiskan Plus microplate reader, and DeltaSoft software.

### 4.3 Aphid propagation

Cultures of apterous *M. persicae* clone US1L (Devonshire & Sawicki, 1979), *B. brassicae*, and *L. erysimi* (Dawson et al., 1987) were kind gifts from Rothamsted Research and were maintained on Chinese cabbage. To obtain aphids of standardized age for use in experiments, 8‐ to 10‐day‐old adult aphids were transferred to aphid‐free Chinese cabbage plants and allowed to reproduce for 24 hr. Newborn nymphs were transferred to individual experimental plants (one aphid per plant) using fine paintbrushes and confined on plants using microperforated plastic bags (Associated Packaging). MRGR and colony growth were assayed as previously described and repeated at least two times (Rhee et al., 2020; Stewart et al., 2009; Tungadi et al., 2020; Westwood et al., 2013; Ziebell et al., 2011). In some experiments, after being weighed, the aphids were placed back on plants and at 14 days after placement the offspring produced by each aphid were counted. Statistical analyses were conducted in R v. 3.5.0 (R Core team, 2014).

## CONFLICT OF INTEREST

The authors declare that they have no conflict of interest.

## AUTHOR CONTRIBUTIONS

S.C.G., T.T., L.G.W., Z.D., J.H.W., G.P., and J.P.C. conceived and designed the experiments. T.T., L.G.W., S.C.G., A.E.P., Z.D., J.H.W., and T.G.F. performed the experiments. T.T., S.C.G., L.G.W., G.P., A.M.M., and J.P.C. analysed the data. S.C.G., T.T., A.M.M., and J.P.C. wrote the manuscript.

## Supporting information

**FIGURE S1** Steady‐state accumulation of cucumber mosaic virus (CMV) in mutant and wild type (WT) *Arabidopsis thaliana* plants. CMV accumulation was measured using a double antibody sandwich enzyme‐linked immunosorbent assay (ELISA) with a primary antibody specific for the CMV coat protein (Bioreba AG). Alkaline phosphatase conjugated to the secondary antibody, catalyses substrate conversion to yellow *p*‐nitrophenol, the accumulation of which was measured spectrophotometrically at 405 nm using a Titertek Multiskan Plus microplate reader and quantified using DeltaSoft software. For each treatment, ELISA was performed separately using extracts from *n* individual plants at 14 days postinoculation or mock inoculation, and error bars represent the standard error around the mean A_405_ value for each treatment. (a) CMV accumulation was compared in WT Col‐0 plants (CMV, *n* = 3; mock, *n* = 2), *bak 1‐5* mutant plants (CMV, *n* = 5; mock, *n* = 2), *bkk 1‐1* mutants (CMV, *n* = 5; mock, *n* = 2), and *bkk 1‐1*/*bak 1‐5* double mutant plants (CMV, *n* = 5; mock, *n* = 2). (b) Comparison of CMV accumulation in WT Col‐0 plants (CMV, *n* = 4; mock, *n* = 3), and *dde 2‐2* mutant plants (CMV, *n* = 3; mock, *n* = 3). (c) CMV accumulation in WT Col‐0 plants (CMV, *n* = 3; mock, *n* = 3) (genetic background of *NahG*‐transgenic plants) and Col‐gl plants (genetic background of *coi1* 1‐16 mutant plants) (CMV, *n* = 3; mock, *n* = 3), *coi 1‐16* (CMV, *n* = 3; mock, *n* = 3) mutants and *NahG* transgenic plants (CMV, *n* = 3; mock, *n* = 3). (d) CMV accumulation if WT Col‐0 plants (CMV, *n* = 4; mock, *n* = 4), *sid 2‐2* (CMV, *n* = 3; mock, *n* = 3), and *ein 2‐1* mutant plants (CMV, *n* = 3; mock, *n* = 3)

**SPREADSHEET S1** Performance of *Myzus persicae* on mock‐inoculated and CMV‐infected *bak1* and *bkk1* mutant plants

**SPREADSHEET S2** Data on aphid performance on *dde2* mutant plants

**SPREADSHEET S3** Data: Aphid performance on *NahG* transgenic and *coi1* mutant plants

**SPREADSHEET S4** Data on aphid performance on *ein2* and *sid2* mutant plants

**SPREADSHEET S5** Data on performance of *Myzus persicae*, *Brevicoryne brassicae* and *Lipaphis erysimi* on mock‐inoculated and CMV‐infected *bak1* and *bak1/bkk1* mutant plants

## Data Availability

All relevant data are within the paper and its Supporting Information files.
