# Supplementary material for: Infection of Arabidopsis by cucumber mosaic virus triggers jasmonate‐dependent resistance to aphids that relies partly on the pattern‐triggered immunity factor BAK1
Source: Mol Plant Pathol. 2021 Jun 22;22(9):1082–91. doi: 10.1111/mpp.13098 (PMC8358999; doi:10.1111/mpp.13098)
Supplement: Supplementary file 1 — FIGURE S1 Steady‐state accumulation of cucumber mosaic virus (CMV) in mutant and wild type (WT) Arabidopsis thaliana plants. CMV accumulation was measured using a double antibody sandwich enzyme‐linked immunosorbent assay (ELISA) with a primary antibody specific for the CMV coat protein (Bioreba AG). Alkaline phosphatase conjugated to the secondary antibody, catalyses substrate conversion to yellow p‐nitrophenol, the accumulation of which was measured spectrophotometrically at 405 nm using a Titertek Multiskan Plus microplate reader and quantified using DeltaSoft software. For each treatment, ELISA was performed separately using extracts from n individual plants at 14 days postinoculation or mock inoculation, and error bars represent the standard error around the mean A405 value for each treatment. (a) CMV accumulation was compared in WT Col‐0 plants (CMV, n = 3; mock, n = 2), bak 1‐5 mutant plants (CMV, n = 5; mock, n = 2), bkk 1‐1 mutants (CMV, n = 5; mock, n = 2), and bkk 1‐1/bak 1‐5 double mutant plants (CMV, n = 5; mock, n = 2). (b) Comparison of CMV accumulation in WT Col‐0 plants (CMV, n = 4; mock, n = 3), and dde 2‐2 mutant plants (CMV, n = 3; mock, n = 3). (c) CMV accumulation in WT Col‐0 plants (CMV, n = 3; mock, n = 3) (genetic background of NahG‐transgenic plants) and Col‐gl plants (genetic background of coi1 1‐16 mutant plants) (CMV, n = 3; mock, n = 3), coi 1‐16 (CMV, n = 3; mock, n = 3) mutants and NahG transgenic plants (CMV, n = 3; mock, n = 3). (d) CMV accumulation if WT Col‐0 plants (CMV, n = 4; mock, n = 4), sid 2‐2 (CMV, n = 3; mock, n = 3), and ein 2‐1 mutant plants (CMV, n = 3; mock, n = 3) [file MPP-22-1082-s003.pdf]

Tungadi et al. Supplementary Figure 1

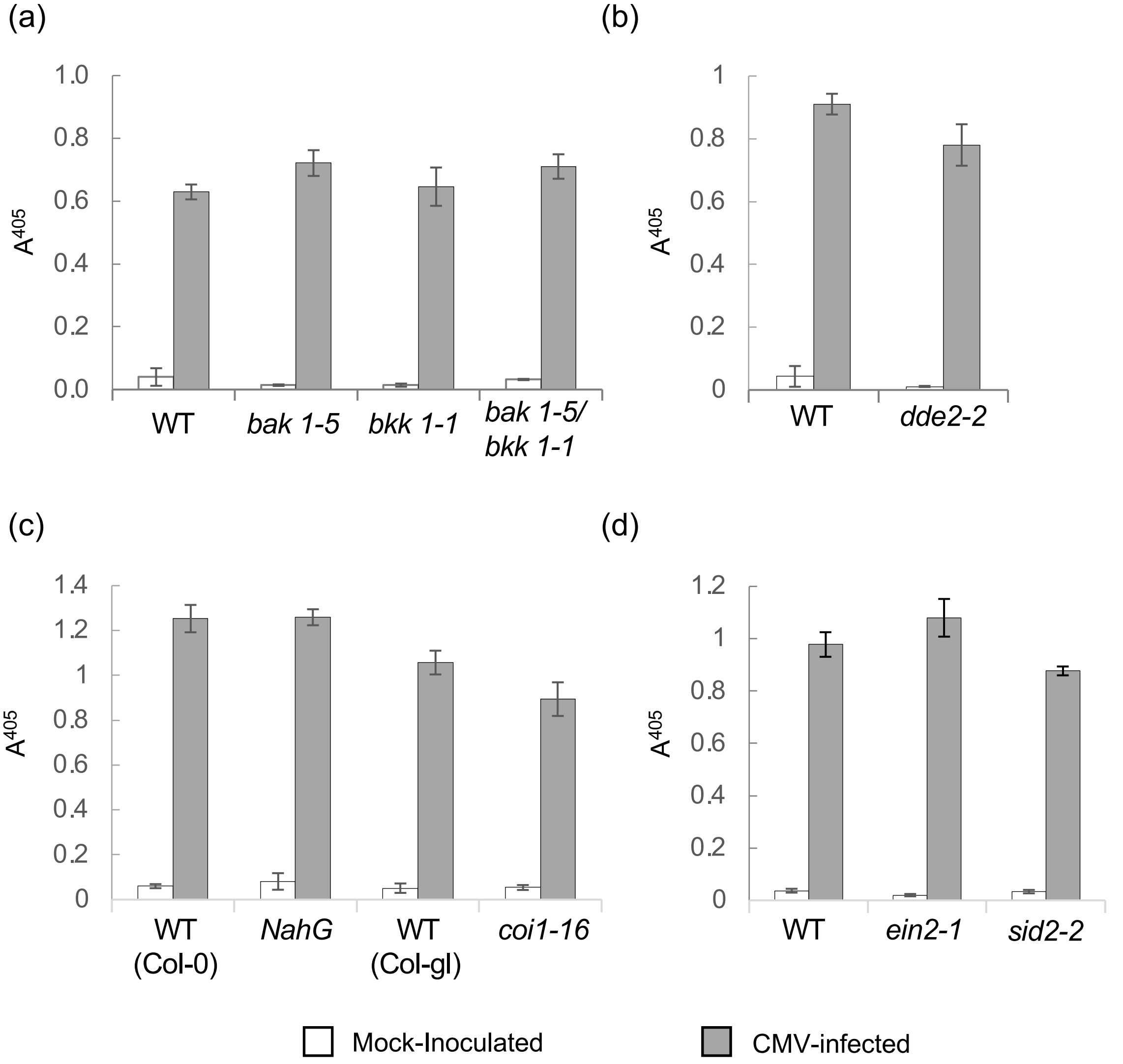

Fig. S1. Steady-state accumulation of cucumber mosaic virus (CMV) in mutant and wild type (WT) *Arabidopsis thaliana* plants. CMV accumulation was measured using a double antibody sandwich enzyme-linked immunosorbent assay (ELISA) with a primary antibody specific for the CMV coat protein (Bioreba AG, Reinach, Switzerland). Alkaline phosphatase conjugated to the secondary antibody, catalyses substrate conversion to yellow *p*-nitrophenol, the accumulation of which was measured spectrophotometrically at 405 nm using a Titertek Multiskan Plus microplate reader, and quantified using DeltaSoft software. For each treatment, ELISA was performed separately using extracts from *n* individual plants at 14 days post-inoculation or mock inoculation, and error bars represent the standard error around the mean A^{405} value for each treatment. (a) CMV accumulation was compared in WT Col-0 plants (CMV, *n*= 3; mock, *n*= 2), *bak 1-5* mutant plants (CMV, *n* = 5; mock, *n*= 2), *bkk 1-1* mutants (CMV, *n*= 5; mock, *n*= 2), and *bkk 1-1*/*bak 1-5* double mutant plants (CMV, *n*= 5; mock, *n*= 2). (b) Comparison of CMV accumulation in WT Col-0 plants (CMV, *n*= 4; mock, *n*= 3), and *dde 2-2* mutant plants (CMV, *n*= 3; mock, *n*= 3). (c) CMV accumulation in WT Col-0 plants (CMV, *n* =3; mock, *n* =3) (genetic background of *NahG*-transgenic plants) and Col-gl plants (genetic background of *coi1* 1-16 mutant plants) (CMV, *n* =3; mock, *n* =3), *coi 1-16* (CMV, *n*=3; mock, *n* =3) mutants and *NahG* transgenic plants (CMV, *n*=3; mock, *n* =3). (d) CMV accumulation if WT Col-0 plants (CMV, *n* =4; mock, *n* =4), *sid 2-2* (CMV, *n*=3; mock, *n* =3), and *ein 2-1* mutant plants (CMV, *n*=3; mock, *n* =3).
